# A rapid prototyped atmospheric non-thermal plasma-activated aerosol device and anti-bacterial characterisation

**DOI:** 10.3389/fchem.2024.1416982

**Published:** 2024-06-14

**Authors:** Jefferson de Oliveira Mallia, Sholeem Griffin, Clara Buttigieg, Ruben Gatt

**Affiliations:** ^1^ Metamaterials Unit, University of Malta, Msida, Malta; ^2^ Centre for Molecular Medicine and Biobanking, University of Malta, Msida, Malta

**Keywords:** rapid prototyped, 3D printed, atmospheric non-thermal plasma, plasma-activated aerosols, anti-bacterial

## Abstract

Non-plasma technologies are being extensively investigated for their potential to mitigate microbial growth through the production of various reactive species. Predominantly, studies utilise atmospheric non-thermal plasma to produce plasma-activated liquids. The advancement of plasma-liquid applications has led to the investigation of plasma-activated aerosols (PAAs). This study aimed to produce a rapid-prototyped plasma-activated aerosol setup and perform chemical and anti-bacterial characterisation on the resultant activated aerosols. The setup was produced using stereolithography 3D printing, and air was used as the carrier gas. The novel design of the device allowed for the direct production of PAAs without the prior generation of plasma-activated water and subsequent aerosolisation. The generated PAAs were assessed for nitrite, hydrogen peroxide and ozone content using colourimetric assays. Anti-bacterial efficacy was tested against three human pathogenic strains: *Escherichia coli*, *Pseudomonas aeruginosa*, *Staphylococcus aureus* and *Salmonella enterica*. It was observed that nitrite and ozone contact concentration increased with exposure time, yet no hydrogen peroxide was detected. The generated PAAs showed significant zones of no growth for all bacterial strains. These devices, therefore, show potential to be used as anti-bacterial disinfection technologies.

## 1 Introduction

Considerable research efforts have been dedicated to exploring the potential of plasma-activated liquids to enhance food quality through the inactivation of harmful food-borne pathogens without the use of undesirable chemicals, as evidenced by several studies (Darmanin et al., 2020; Darmanin et al., 2021; Schnabel et al., 2021). The origins of non-thermal plasma technology can be traced back to the late 20th century (Thirumdas et al., 2015), and unlike traditional thermal plasmas, non-thermal plasma operates at near ambient temperatures, thereby minimising thermal damage (Laroussi, 2002). Non-thermal plasma can be generated at atmospheric pressure by applying a high voltage (corona discharge or dielectric barrier discharges) to a gas stream such as argon, nitrogen, or air. Activation of water using non-thermal plasma results in changes in its physicochemical properties, including electrical conductivity, oxidation-reduction potential and pH (Ma et al., 2015; Zhang et al., 2016). These properties change due to the formation of reactive oxygen and nitrogen species, such as ozone, hydrogen peroxide (He et al., 2018; Lin J. et al., 2020), nitrates and nitrites (Girard et al., 2016; Zhou et al., 2018; Wu et al., 2021), during the activation process. Plasma-activated water (PAW) was found to have good anti-microbial potential with studies being conducted on planktonic, adhered or food-surface-attached cells (Kamgang-Youbi et al., 2009; Sarangapani et al., 2018; Thirumdas et al., 2018). In such studies, it was determined that microbial inactivation was a result of the action of reactive oxygen and nitrogen species present in PAW. Furthermore, it was shown that a higher anti-microbial activity was observed with an increase in the oxidative-reductive potential and a decrease in the pH of the activated solution (Zhang et al., 2016; Thirumdas et al., 2018). In this regard, non-thermal atmospheric plasma has been investigated to meet the needs of physical decontamination technologies in the health, waste management and food sectors.

The production of large volumes of activated liquids presents a set of practical challenges, encompassing issues related to production time and stability when stored (Wang and Salvi, 2021; Chew et al., 2022). These challenges have spurred the exploration of alternative solutions to circumvent the limitations associated with using activated liquids. One such alternative approach which is gaining attention is the utilisation of aerosols that can be activated through non-thermal plasma (Stancampiano et al., 2019). Converting small volumes of liquids into aerosols can be accomplished through various techniques, such as high-frequency ultrasound waves or pressurised gas streams. The resulting aerosol mixtures still retain liquid micro-droplets. As stated by Stancampiano et al., a plasma-aerosol consists of a gas dispersing a dynamic suspension of liquid droplets within a plasma (Stancampiano et al., 2019). The advantage of this configuration is that it enables enhanced activation energy transfer and more control of liquid reactivity and production parameters.

Recent advancements in this field have showcased the feasibility of utilising PAW, which can be nebulised using acoustic waves, for bactericidal applications (Wong et al., 2020). Several studies have demonstrated the enhanced anti-microbial properties of plasma-activated hydrogen peroxide aerosols (Freyssenet and Karlen, 2019; Song et al., 2020). Chew and co-workers (Chew et al., 2023) have shown synergistic effects when combining aerosolisation with atmospheric pressure plasma generation. They found that pretreating stainless-steel surfaces with a hybrid spray-deposition technique before pathogen exposure resulted in almost complete inactivation of the bacteria colonies. In another study by Chew et al., it was shown that it was possible to miniaturise a system capable of producing plasma-activated aerosols for surface disinfection (Chew et al., 2022).

This study investigated a rapid-prototyped plasma-activated aerosols (PAA) setup for its chemical and anti-bacterial properties. In contrast to other studies (Wong et al., 2020; Chew et al., 2022; Woo et al., 2023; Chew et al., 2024), this study reports on the direct production of PAAs and the chemical changes induced to aerosols without the prior generation of plasma-activated water. Further to other studies (Freyssenet and Karlen, 2019; Song et al., 2020; Song and Fan, 2020; Fan et al., 2022), this study does not include the addition of hydrogen peroxide to induce anti-microbial effects. This study reports the production of pH, hydrogen peroxide, nitrite and ozone content produced within PAAs. This is to provide a better understanding of the chemical changes occurring between the aerosol and non-thermal atmospheric plasma discharge, focusing on interactions between the reactive species observed and quantifying their resultant long-lived chemical products. The device was then utilised to assess its surface disinfection capacity against human pathogenic bacterial strains. This is to further our understanding of the chemical interactions occurring between aerosols and non-thermal atmospheric plasma and the potential PAA technologies have towards their application as anti-bacterial disinfection technologies.

## 2 Materials and methods

### 2.1 Design and fabrication of the plasma-activated aerosol parts

Components that formed the plasma-activated aerosol setup were designed using Autodesk^®^ Inventor^®^ Professional 2023 (Build: 158). The reactor parts were then fabricated using stereolithography (Formlabs). The printer had an xy resolution of 50 μm, and a layer thickness of 50 μm was utilised. Parts in the vicinity of the generated plasma were printed using high-temperature resin, whilst other parts of the device were printed using Though 2000 resin. After the printing process, the prototypes underwent a wash in isopropyl alcohol (IPA) within the Formlabs Form Wash tank (10 min in the case of the Though 2000 resin and 5 min in the case of the High-Temperature resin). Subsequently, the parts were separated from the build plate, eliminating any support material. Following this, the cleaned models underwent an additional wash in IPA (10 min for the Though 2000 resin and 5 min for the High-Temperature resin). Finally, the washed prototypes were post-cured using the Formlabs Form Cure at 60 °C for 60 min in the case of the Though 2000 resin and at 70 °C for 120 min in the case of the High-Temperature resin. A number of supporting parts were also produced using a 3D Fused Deposition Modelling (FDM) printer (Creality) employing a 1.75 mm polylactic acid filament.

### 2.2 Plasma-activated aerosol setup and operation

**FIGURE 1:**
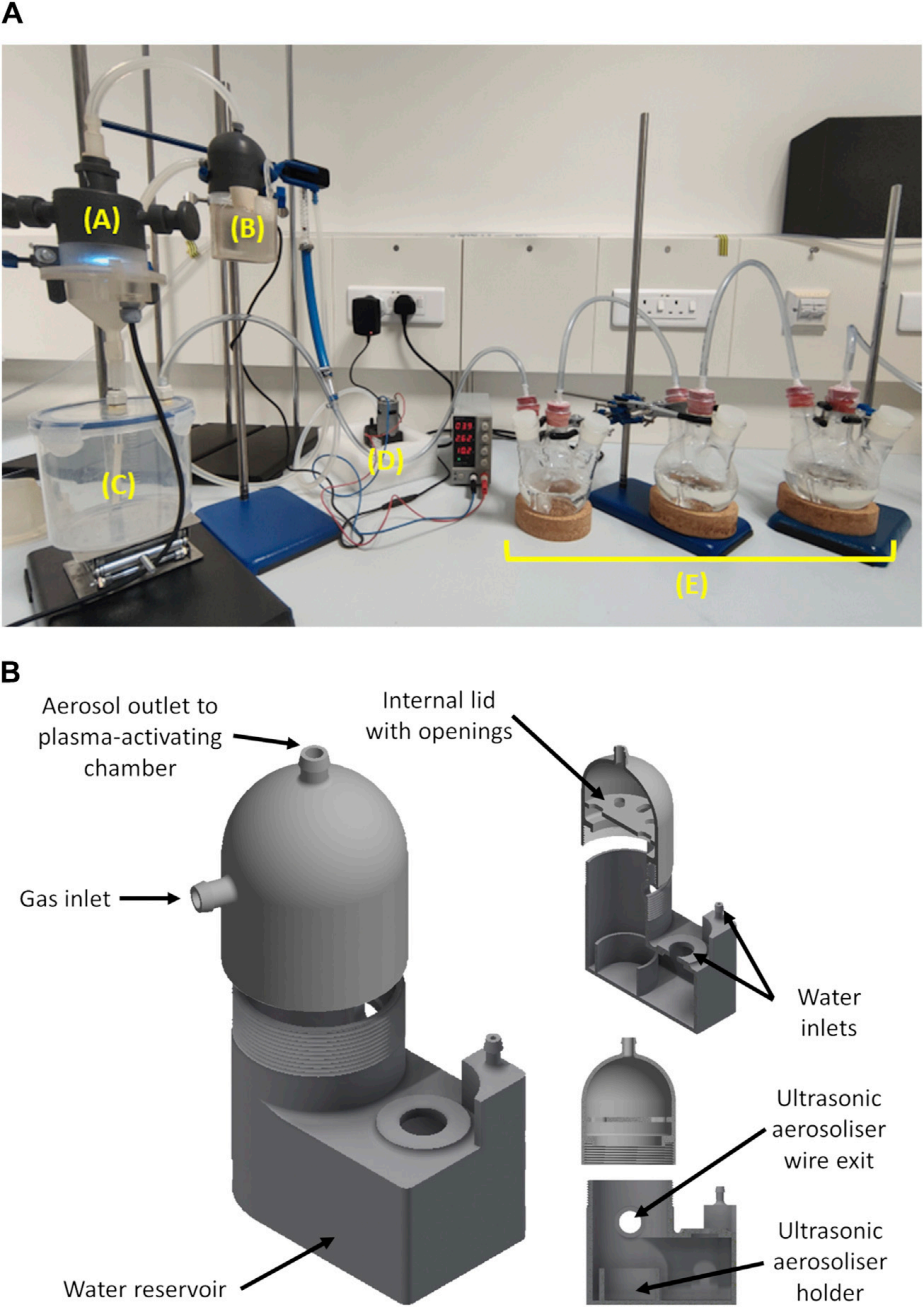
A photo **(A)** of the developed plasma-activated aerosol setup in operation showing the plasma-activating chamber **(A)**, aerosol generating chamber **(B)**, aerosolizing chamber **(C)**, air pump **(D)** and bubbling flasks **(E)**. The schematic diagram **(B)** shows the aerosol generating chamber with cross sections showing the internal compartments.

The device was operated by going through a series of steps. The first step required the addition of water in the aerosolising chamber (component B in Figure 1). It was ensured that the water level always submerged the ultrasonic aerosoliser unit. The proposed unit consists of a piezo ceramic disc that was connected to a high-frequency signal generator and a power supply unit that generates high-frequency mechanical vibrations. The focused ultrasonic waves initiate cavitation that forms water particles. The suspension of these particles in a selected gas generated the aerosol. The desired gas, air in the case of this study, was pumped into an aerosol chamber using a gas pump (VN-C4, You Cheng Industrial Co. Ltd.). The pump drew room air and circulated it through the setup at a set gas flow rate. Prior to entering the aerosol-generating chamber, the gas passed through an in-line 0.22 µm PTFE filter. A high-voltage power supply and controller unit (HY-Z150, Cloudray) was used to provide the potential difference across the copper electrodes housed within the plasma activating chamber (component A in Figure 1) that generated the non-thermal plasma. All experiments reported in this study were done at a flow rate of 150 L/h and a power supply control voltage of 0.4 V, which translated to an average potential difference of circa 30 KV and an average current of circa 0.1 mA. At this point, the produced PAA passed through an aerosolising chamber (component C in Figure 1). The gas from the aerosolising chamber was then bubbled through a series of flasks containing 250 mL of deionised water. The various components that form the system are shown in Figure 1.

### 2.3 Optical emission spectra (OES) acquisition

An optical emission spectrophotometer (Ocean Optics) was used to acquire OES spectra. Spectra were processed using the OceanView software (Version 1.6.5). A special holder was designed and 3D printed using high-temperature resin to hold a collimating lens that was unobstructed and proximate to the plasma discharge. This was then connected to the spectrophotometer via a fibre optic cable.

### 2.4 Rate of aerosol formation

The rate of aerosol formation was measured as follows. The aerosol-generating chamber was filled with ultra-pure water, ensuring full submersion of the ultrasonic aerosoliser unit. The total mass of the filled aerosol-generating chamber was then measured using a KERN PLJ 2000-3A mass balance with a readability of 0.001 g and a linearity of ±0.004 g. Subsequently, the aerosoliser unit was activated, and air was passed through the aerosol-generating chamber at a rate of 150 L/h for a duration of 5 min. Following this, the ultrasonic aerosoliser unit was switched off, and the mass of the aerosol-generating chamber was re-measured. This process was repeated for time intervals of 10, 15, 20, and 25 min. Linear regression was performed to obtain Eq. 1, which shows the mass of aerosol produced as a function of time.

$$M_{aero}=58.54t-27.71$$ (1)

where *t* is the time lapsed, and *M*_*aero*_ is the mass of aerosol produced during that time interval.

### 2.5 Chemical characterisation

The generated PAAs were assessed by bubbling the released gas, leaving the aerosolising chamber through a series of flasks containing 250 mL of deionised water (Milli-Q, Thermo Fisher). Deionised water samples were collected and chemically assessed.

#### 2.5.1 pH measurements

The pH values reported during this study were measured using a pH meter (Hanna HI-2020-02, Hanna Instruments). Then Eq. 2 was used to obtain the aerosol pH (pH_aero_) from that determined in bubbling flask solutions.

$${\text{pH}}_{aero}=\log\left(\frac{M_{aero}}{D_{water}.V}\right)+{\text{pH}}_{sol}$$ (2)

where *M*_*aero*_ (g) is the mass of aerosol produced for a specific time interval, *D*_*water*_ (g.mL^-1^) is the density of water, *V* (mL) is the volume of deionised water in a bubbling flask, and pH_sol_ is the pH measured for the bubbling flasks.

#### 2.5.2 Nitrite quantification

The nitrite content in PAA was determined using a Griess colourimetric assay as described in Murray et al. (Murray et al., 2017) and Darmanin et al. (Darmanin et al., 2020) with some modifications. Briefly, the sulphanilamide reagent was prepared by dissolving 2.5 g of sulphanilamide (Biochem Chemopharm), in 25 mL of 37% hydrochloric acid (Carlo Erba) and diluting this to 250 mL with deionised water. The N-(1-naphthyl)-ethylenediamine dihydrochloride (NED, Biochem Chemopharma) reagent was prepared by dissolving 0.25 g of NED and diluting this to 250 mL with deionised water. The Griess detection reagent was prepared by mixing the sulphanilamide and NED reagents in a 1:1 volume ratio. Standard solutions of known nitrite concentrations were prepared using sodium nitrite (Biochem Chemopharma). The determination of nitrite in both standard and sample solutions was done by a 1:10 ratio of detect reagent to sample or standard and incubating for 5 min in 96-well plates (Thermo Fisher). The absorbance was then measured at 540 nm using a plate reader spectrophotometer (Clariostar, BMG). Then Eq. 3 was used to obtain the aerosol concentration of nitrite (*C*_*aero*_) from that determined in bubbling flask solutions.

$$C_{aero}=\frac{C_{sol}.V}{D_{air}.F.t}$$ (3)

where *C*_*sol*_ (ppm) is the concentration of nitrite in the bubbling flasks, *V* (mL) is the volume of deionised water in bubbling flasks, *D*_*air*_ (g.L^-1^) is the density of air, *F* (L/min) is the air flow rate and *t* is the time lapsed.

#### 2.5.3 Hydrogen peroxide quantification

The hydrogen peroxide (H_2_O_2_) content was determined using a colourimetric titanium sulphate (TiSO_4_) as described in Eisenberg (Eisenberg, 1943) and Darmanin et al. (Darmanin et al., 2020) with some modifications. In brief, 2 g of anhydrous titanium dioxide (Biochem Chemopharma) was transferred in 200 mL of 96% sulphuric acid (Scharlau). The mixture was then heated up to 150°C and left for at least 15 h. The mixture was then cooled down to room temperature and diluted in deionised water with a 1:3 volume ratio. The diluted mixture was filtered through a sintered glass funnel (Grade/Porosity four or higher). The resultant filtrate was the detection reagent. Standard solutions of known H_2_O_2_ concentrations were prepared using 30% wt. H_2_O_2_ (Scharlau). This was done by adding a 1:4 ratio of detect reagent to sample or standard for 5 min in 96-well plates. The absorbance was then measured at 410 nm using a plate reader spectrophotometer (Clariostar, BMG).

#### 2.5.4 Ozone quantification

The ozone (O_3_) produced was initially detected using an ozone detector (Draeger). It was quantified using a colourimetric potassium iodide assay as described in Shechter (Shechter, 1973) and Sugita et al. (Sugita et al., 1996) with some modifications. In brief, a pH seven phosphate buffer was prepared by dissolving 8.0 g of sodium chloride (Biochem Chemopharma), 0.2 g of potassium chloride (Biochem Chemopharma), 1.15 g of disodium hydrogen phosphate (Biochem Chemopharma) and 0.2 g of potassium dihydrogen phosphate (Biochem Chemopharma) in 1 L of deionised water. The pH was then adjusted with 1 M potassium hydroxide (Carlo Erba) or 1 M orthophosphoric acid (Fisher Scientific) to pH 7. A solution of 2% (w/v) potassium iodide (Carlo Erba) was then prepared using the pH seven phosphate buffer. The resultant solution was the detection reagent. Standard solutions of known iodine (I_2_, Carlo Erba) concentrations were also prepared. Quantification was done by adding a 1:1 ratio of detected reagent to sample or standard, mixing and incubating for 40 min in 96-well plates. The absorbance was then measured at 350 nm using a plate reader spectrophotometer (Clariostar, BMG). The concentration of I_2_ as triiodide represented the concentration of ozone detected. The aerosol concentration of ozone was determined using Eq. 3, using the concentration of ozone determined in the bubbling solutions instead.

### 2.6 Anti-bacterial characterisation

**FIGURE 2:**
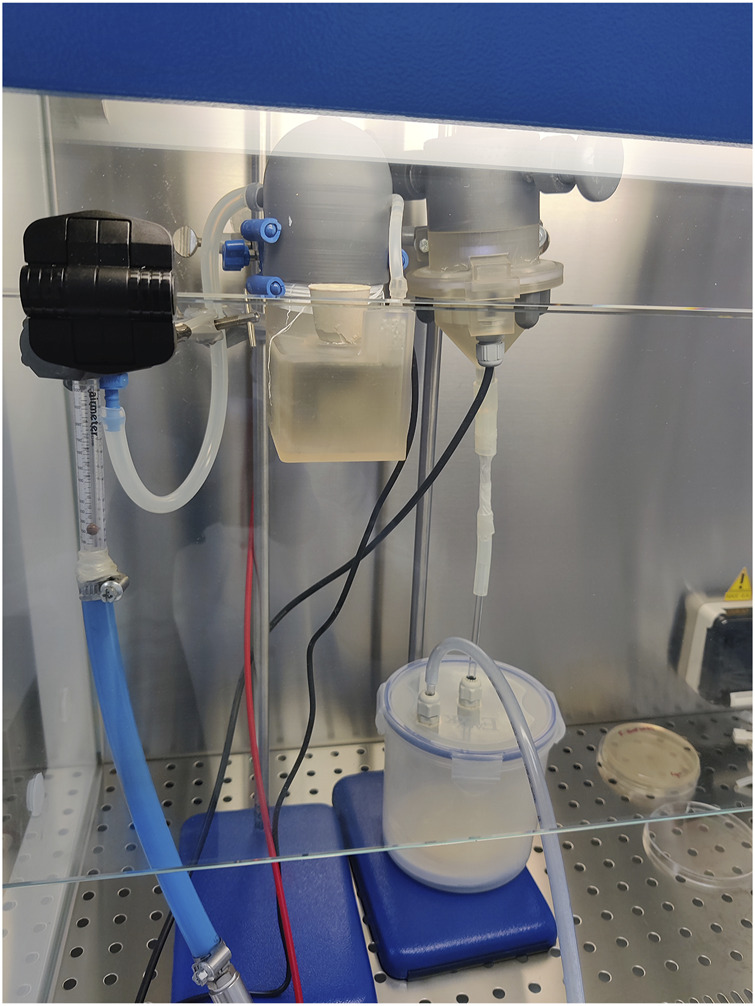
Anti-bacterial PAA testing of setup within BSL2 A2 cabinet.

The methods for assessing the anti-bacterial efficacy of PAAs utilised human pathogenic strains of *Escherichia coli K12* (NCTC 10538), *Pseudomonas aeruginosa* (ATCC 15442), *Staphylococcus aureus* (ATCC 6538) and *Salmonella enterica* serovar Abony (NCTC 6017). All tests were carried out under aseptic conditions in a BSL2 A2 cabinet (Faster Srl), as shown in Figure 2.

Test samples were prepared by inoculating a single colony of the test microorganism in tryptic soy broth (Oxoid) and incubated overnight in a 37°C incubator (VELP Scientifica Srl) in a shaker rotating (Grant Instruments) at 200 rpm. The resultant bacterial suspension was centrifuged and resuspended in fresh tryptic soy broth. Standard experiments were carried out to enumerate these suspensions containing the test microorganisms to standardise the number of colony-forming units (CFU) per test plate by plate counting technique. A 10^4^ CFU mL^-1^ dilution of the test suspension was made and applied using an L-shaped spreader to a tryptic soya agar medium plate so that the test organisms were spread on a diameter of 9 cm. The plates were then placed in the sealed aerosolising enclosure directly under the aerosol outlet of the plasma device to access the bactericidal activity of the PAAs. The test plate containing microorganisms was in contact with aerosols for 15 min, after which it was covered and placed in an incubator set at 37°C for 18 h. The plate was inspected visually to assess disinfection. The area of no growth on the Petri dish after exposure to PAAs was measured so that two measurements were taken per plate, one horizontally and one vertically. This was to obtain an average diameter of the zone of no growth. Similarly, untreated plates were also measured. However, the zone of growth in this was always 9 cm.

### 2.7 Data and statistical analysis

Data was analysed using GraphPad Prism 8.0.1. Adherence of data was tested with the Anderson-Darling and D'Agostino-Pearson omnibus test. A One-way Analysis of Variance with Dunnett’s *post hoc* multiple comparisons test was carried out for data adhering to a Gaussian distribution. The alpha was set to 0.05.

## 3 Results

### 3.1 Observed OES spectra

**FIGURE 3:**
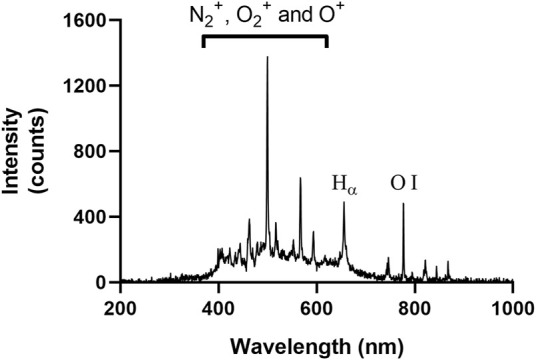
The OES spectra acquired during PAA production when using air.

The OES spectra were acquired during PAA production, as shown in Figure 3. The most predominant bands within the OES spectra for air were obtained between 400 and 600 nm due to the presence of first negative system for N_2_^+^ O_2_^+^ and O^+^ species (Shin et al., 2008; Naz et al., 2021). Characteristic emissions were also observed for atomic hydrogen (Hα, 656 nm), nitrogen (747, 822, and 868 nm) and oxygen (O I, 777 and 844 nm) (Ma et al., 2017; Thana et al., 2019).

### 3.2 Rate of aerosol formation

**FIGURE 4:**
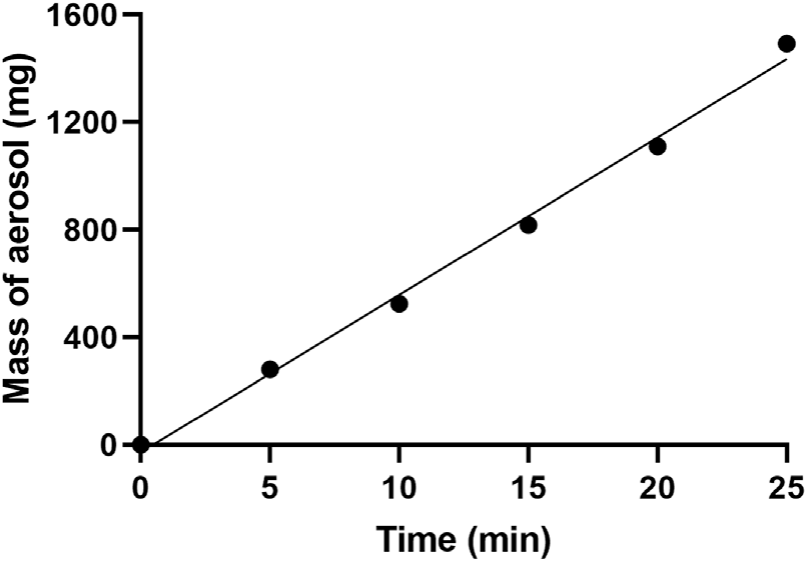
Plot showing the mass of aerosol formation at different time intervals. The *R* ^2^ for the line of best fit was found to be 0.9951.

The mass of water that exited the aerosol-generating chamber at different time intervals was determined and is depicted in Figure 4. The assumption was made that all the water exiting the chamber was in aerosolised form (an assumption supported by observation), and the rate of aerosol formation was calculated from the slope of this plot as 57.03 mg/min.

### 3.3 Chemical characterisation

#### 3.3.1 Variation of pH

**FIGURE 5:**
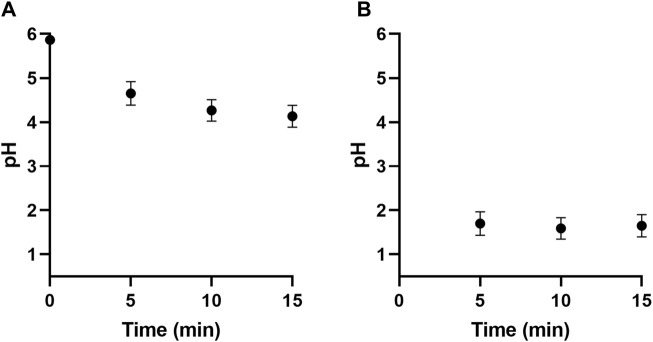
The change in pH in deionised water samples contained within the first bubbling flask with increasing PAA exposure **(A)**. The pH of the PAA was then determined **(B)**. Data is presented as the mean (±S.D.) of three independent runs (*n* = 3).

The pH was measured at different time intervals in the first trapping flasks. The measurements which were obtained are shown in Figure 5A. It was observed that after 15 min of production, the pH in the first trapping flasks was circa 4.2. The pH of the PAA was then determined, as shown in Figure 5B. It was determined the aerosol pH at the end of the test period was about 1.6.

#### 3.3.2 Variation of nitrite and hydrogen peroxide quantification

The hydrogen peroxide and nitrite content were determined using titanium sulphate and Griess colourimetric assays, respectively. In all cases, the contents of all trapping flasks were first added together and mixed thoroughly. In the case of this study, hydrogen peroxide was not detected in any solution at any time interval. Nitrite content in the bubbling flasks at different time intervals is shown in Figure 5A. The nitrate content within the PAAs was then determined, as shown in Figure 5B. It was observed that nitrite content within the PAAs was about 20 ppm at the end of the test period.

#### 3.3.3 Ozone variation

**FIGURE 6:**
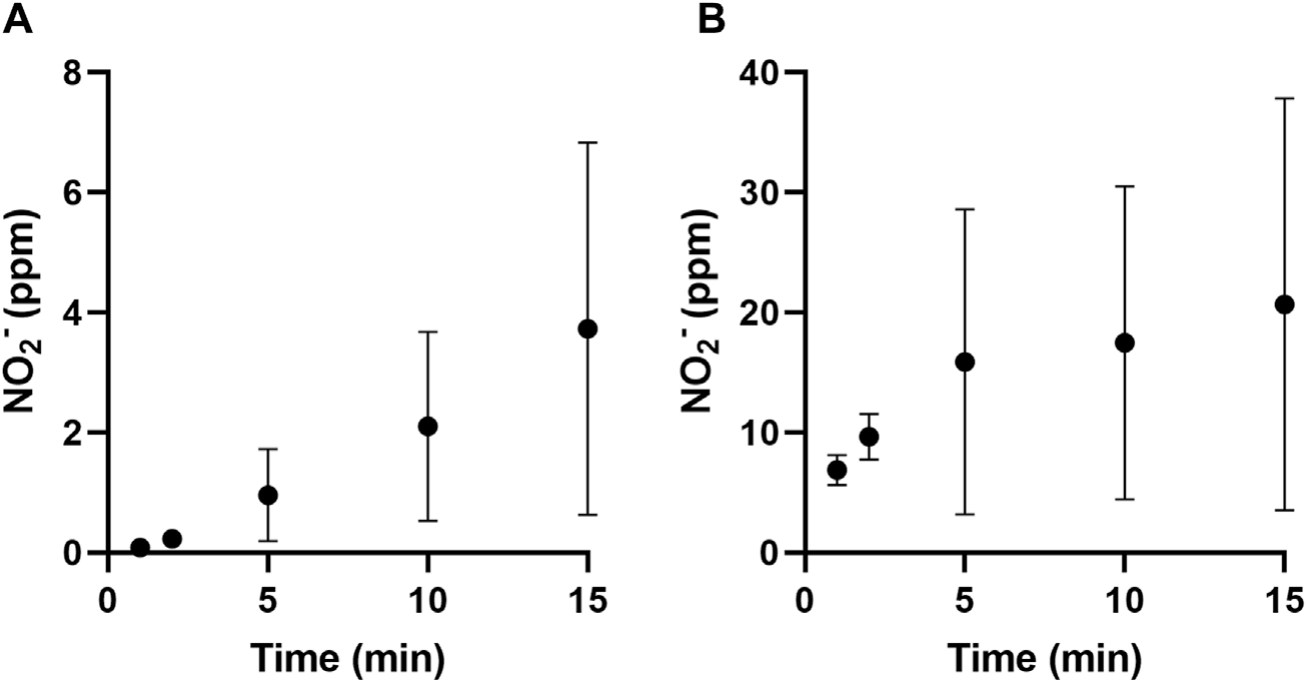
The nitrite content in the bubbling flasks **(A)** and that contained in the PAAs **(B)** at different time intervals. Data is presented as the mean (±S.D.) of three independent runs (*n* = 3).

The presence of ozone generated by the PAA was initially confirmed by an ozone detector. Ozone was quantified using a KI assay that oxidised iodide into iodine, which could be quantified through the formation of triiodide. This generated a yellow colour that increased in intensity with longer PAA exposures. The contents of all three trapping flasks were added together and mixed thoroughly before analysis. The measured ozone concentration at different time intervals is shown in Figure 6A. The ozone content within the PAAs was then determined, as shown in Figure 6B. It was observed that nitrite content within the PAAs was about 21 ppm at the end of the test period.

### 3.4 Anti-bacterial efficacy testing

**FIGURE 7:**
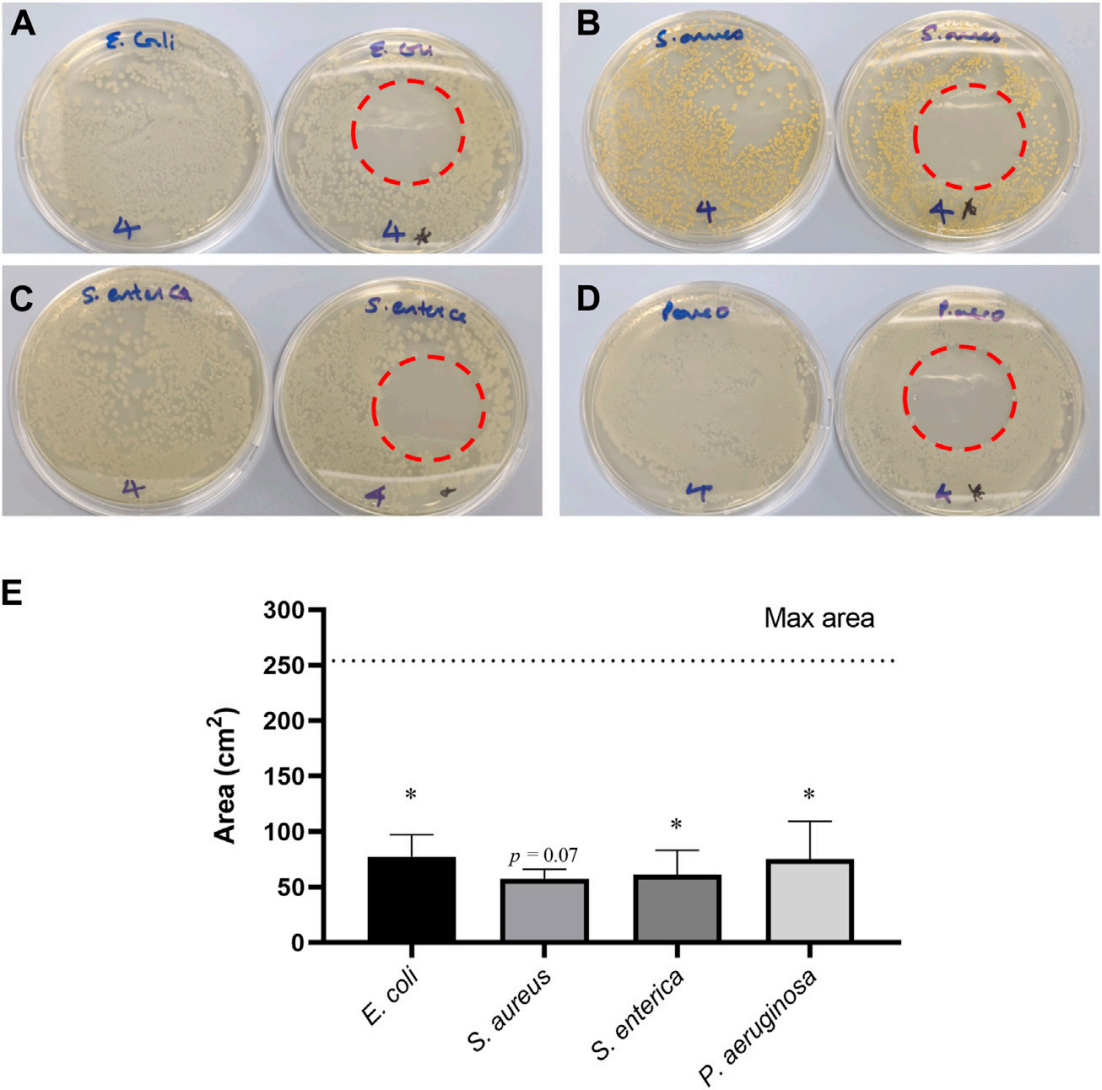
A photographic representation of the plates obtained for *Escherichia coli* **(A)**, *S. aureus* **(B)**, *S. enterica* **(C)** and *Pseudomonas aeruginosa* **(D)**, showing a control plate (left) and the no growth zone obtained on a plate that was directly exposed to PAAs for 15 min (right). Zones of no growth **(E)** after direct exposure to PAAs for 15 min were significant. Data is presented as mean (±S.D.) for two biological replicates (*n* = 2). Statistical comparison was done between samples means and untreated control (max area), where significant data is marked with *p ≤ 0.05. When 0.05 < *p* ≤ 0.1, value is indicated.

The direct exposure of PAAs to *E. coli*, *P. aeruginosa*, *S. aureus* and *S. enterica* significantly decreased the area of growth of each tested microorganism relative to the maximal growth of the respective microorganism. Each area of no growth corresponded to a 3-log reduction, which is statistically significant. However, the complete plate did not show reduced growth. This resulted in a halo of complete no growth in the centre of the plate directly below the aerosol outlet, as shown in Figures 7A–D. A statistically significant reduction in the area of microbial growth was observed, where 30.6%, 22.7%, 24.2% and 29.7% for *E. coli*, *S. aureus, S. enterica* and *P. aeruginosa,* respectively, of the plate had no growth as shown in Figure 7E.

## 4 Discussion

Several studies have demonstrated the potential of plasma-activated aerosols (PAAs) for disinfection (Jiang et al., 2017; Freyssenet and Karlen, 2019; Song et al., 2020; Song and Fan, 2020; Wong et al., 2020; Chew et al., 2022; Chew et al., 2023). It was observed that most PAA setups utilised commercial aerosol-generating setups, yet none of the PAA setups utilised 3D printing for additive manufacturing and rapid prototyping. The use of 3D printing in the rapid prototyping of non-thermal plasma setups has been previously reported (Kanazawa et al., 2015; Roszkowska et al., 2023) and allowed for greater flexibility in modifying these setups to improve the chemical assessment of plasma-activated liquids. The studies emphasised that the use of fused deposition modelling (FDM) 3D printing was critical in the iterative development of these non-thermal plasma reactors due to their relative ease of use, versatility and cost-efficiency. In our study, stereolithography (SLS) 3D printing was used to fabricate the main components for the PAA reactor and FDM was used for other supporting components. This allowed for the precise and tailored fabrication of the parts (Figure 1B) to improve device operation via better control of aerosol generation and transfer to the non-thermal plasma reaction chamber. The study by Roszkowska et al. reported extensive degradation of methylene blue that was attributed to the interaction of OH radicals, as observed from OES spectra (308 nm peak) and the resultant formation of H_2_O_2_. In this study, however, this was not observed in OES spectra (Figure 3), and H_2_O_2_ was below the limits of detection limit. The absence of the OH peak in the OES spectra and that of hydrogen peroxide further indicates that water particles and OH radicals are not interacting sufficiently at the liquid gas interface. Hydrogen peroxide formation is initiated by OH radicals (Lin J. et al., 2020; Chen et al., 2020). It was reported by Liu et al., that longer distances between electrodes would result in stronger charge overflow that causes higher rates of dissociation of water and oxygen molecules (Liu et al., 2014). This resulted in higher concentrations of OH and atomic oxygen. The design of the fabricated PAA device could have prohibited the formation of OH and, consequently, hydrogen peroxide due to the short distance between discharge electrodes. This is an important consideration when fabricating non-thermal plasma prototype systems, as design aspects related to the orientation of the discharge electrodes directly influence the reactive species formed and their long-lived chemical species.

Studies that assessed aerosols produced from plasma-activated water (Wong et al., 2020; Chew et al., 2022) reported resultant aerosols with pH values between 3.5 and 4.75 and nitrite concentrations between 2 ppm and 10 ppm, compared to pH 1.6 and 20 ppm as observed in this study. Both studies transferred PAW to the surface acoustic wave aerosoliser via a paper strip that acted as a porous conduit. The transport of PAW through this medium could have affected the pH and nitrite content of the final aerosol via retention within this porous matrix. In contrast to this study, the direct transfer of aerosol to the non-thermal plasma reaction chamber to form PAA without any transferring medium showed increased acid and nitrite content in the assessed aerosols. Furthermore, it was observed that the non-thermal plasma activation section of the device reported by Wong et al. and Chew et al. was not enclosed and could have resulted in the loss of reactive species from being retained by the liquid. The complete enclosure of the device reported in this study improved the interaction between aerosol and reactive species generated, increasing the amount of long-lived chemical species of nitrite in the PAA. This could have improved the absorption of the gaseous, long-lived chemical species of ozone in the PAA droplets. Other studies have reported on the non-thermal plasma activation of aerosols within enclosed devices with increased ozone and nitrite content (Gao et al., 2023; Upadrasta et al., 2023). Still, these studies heavily relied on prefabricated materials for their various compartments and did not report using 3D-printed rapid prototyping to fabricate their devices. The study by Gao et al. reported on a recirculation PAA device that produced PAAs containing about 17 ppm of nitrites, which is similar to that reported here. The other study by Upadrastra et al. reported 77 ppm of nitrite and less than 1 ppm of ozone content in PAAs generated by two sequential non-thermal plasma jets. The higher nitrite concentrations can be justified by increased interactions between reactive nitrogen species and the aerosol due to the dual sequential jet setup. The low ozone quantities were attributed to its interaction with other chemical species, such as nitrite.

Further assessment of the acquired OES spectra (Figure 3) enabled us to characterise the reactive species interacting with the aerosol inside the reaction chamber. It was noted that no prominent OH peak appeared at 308 nm (Hong et al., 2012). Furthermore, the characteristic peaks of the first and second positive band system of nitrogen between 400-500 nm and 600–800 nm, respectively, were not clearly observed (Shin et al., 2008). These are typically observed in non-thermal plasma OES spectra containing nitrogen. A recent study by Lamichhane et al. reported that the distance between the non-thermal plasma discharge and the OES optical fibre changes the OES spectra acquired (Lamichhane et al., 2022). It was reported that the peaks for positive systems of nitrogen decreased in intensity with distance. The optical fibre in the PAA setup was located in the middle of the plasma discharge electrodes, so it was not the factor that contributed to the nitrogen-positive systems not appearing. Furthermore, peaks were observed at 400–800 nm, which was due to the first negative system of the positively charged nitrogen species (N_2_^+^) molecules. Peaks could also be associated with emissions coming from O_2_^+^ and O^+^ species (Naz et al., 2021). The presence of nitrite in the PAA aerosol indicated that there are reactions occurring between reactive oxygen and nitrogen species present (Lamichhane et al., 2020; Keris-Sen and Yonar, 2023). The formation of nitrite is initiated by the reaction of atomic nitrogen (747, 822, and 868 nm) with OH. This could indicate that atomic nitrogen consumes any OH radicals to form nitric oxide (NO), releasing hydrogen radicals. Another pathway for the formation of NO radicals is via the combination of nitrogen and oxygen radicals (Keris-Sen and Yonar, 2023). Nitric oxide causes the formation of nitrous acid when dissolving in the aerosol droplets, forming nitrite, as reported in Figure 6.

From the anti-bacterial studies shown in Figure 7, it was observed that the prototype PAA setup was still effective in showing considerable bactericidal surface effects. A 3-log reduction was observed for *E. coli, S. aureus, S. enterica and P. aeruginosa* bacteria; the area of no growth was not statistically significant for *S. aureus*. This may be attributed to the thicker peptidoglycan cell wall of the Gram-positive *S. aureus* bacterium that provides it with more physical protection against reactive oxygen species when compared to Gram-negative *E. coli, S. enterica and P. aeruginosa* bacteria (Moore et al., 2000; Zhang et al., 2023). The combined presence of low pH, ozone and nitrite is most likely responsible for the observed effects and is known to correlate with higher anti-bacterial activities in plasma-activated water (PAW) (Lin C.-M. et al., 2020; Hou et al., 2021). It has been reported that low pH of plasma-activated substances causes cell membrane impairment via damage to phospholipids. This causes increased cell membrane permeability that results in higher cell leakage and infiltration of extracellular substances. The combined anti-bacterial effects of nitrite and low pH combined with that of ozone could have further increased the bactericidal capacity of PAAs as the constant and fixed decomposition of the aerosol would have formed a liquid layer on top of the inoculated agar plates mimicking the anti-bacterial effect of PAW.

**FIGURE 8:**
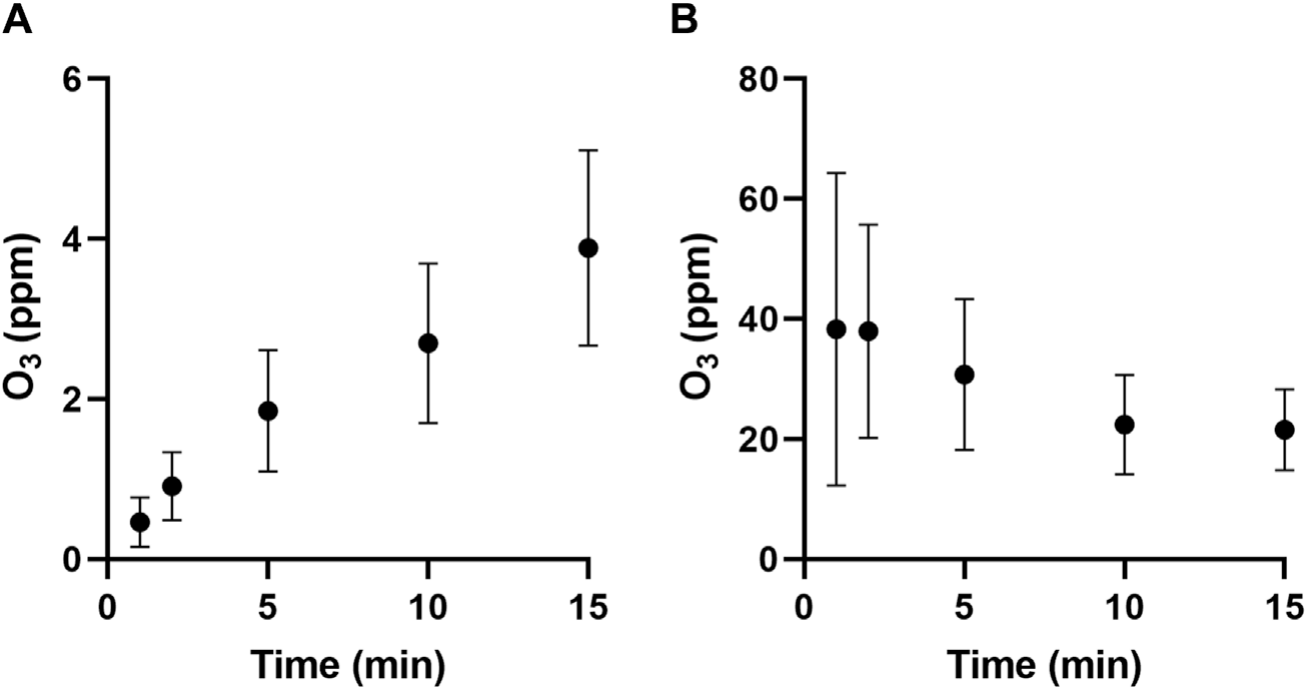
The ozone content in all bubbling flasks **(A)** and that contained in the PAAs **(B)** at different time intervals. Data is presented as the mean (±S.D.) of three independent runs (*n* = 3).

Studies have been conducted and showed that PAAs have significant bactericidal surface effects against *S. enterica serovar Typhimurium* (Jiang et al., 2017; Song et al., 2020), *E. coli* O157:H7 (Wong et al., 2020) and *E. coli BL21* (DE3) (Wong et al., 2020; Chew et al., 2023). Bacterial inactivation in these studies was attributed to the presence of hydrogen peroxide and nitrite in aerosols. It is well known that these can react together to form peroxynitrite, which is a highly reactive anti-microbial agent (Majou and Christieans, 2018; Keris-Sen and Yonar, 2023). Its strong oxidising and nitrating capacity give it the ability to attack a variety of biomolecules, such as lipids and DNA, that lead to the impairment of cellular functionality (McLean et al., 2010; Naitali et al., 2012). In the absence of hydrogen peroxide, as observed in this study, peroxynitrite can still be formed via the intracellular reaction between nitric oxide and anionic superoxide radicals (Goldstein and Merényi, 2008; Hughes, 2008). Nitric oxide is produced via the reduction of internalised nitrite due to bacterial reductases (Besson et al., 2022). Superoxide radicals are produced by the autoxidation of enzymes containing flavoproteins due to the interaction of molecular oxygen and dihydroflavin (Imlay, 2013; Fasnacht and Polacek, 2021; Seixas et al., 2022). It is important to note that the autoxidation of flavoproteins can also cause the production of endogenous hydrogen peroxide, which provides another route for the formation of peroxynitrite. The low pH conditions reported in this study further contributed to bactericidal effects, as studies have shown that spontaneous bacterial inactivation due to acid stress mostly occurs below pH three in various matrices (Tanner and James, 1992; Ikawa et al., 2010; Burin et al., 2014). The low pH conditions could have promoted the formation of intracellular hydroperoxyl radicals through the reaction of protons and superoxide, inducing further oxidative stress damage. It has been shown that hydrogen peroxide, in combination with acidified nitrite, results in a synergistic anti-bacterial effect by promoting the formation of peroxynitrite (Kono et al., 1994; Heaselgrave et al., 2010). Ozone is an extensively studied oxidative agent, and its anti-bacterial efficacy varies among different microorganisms (Komanapalli and Lau, 1998; Moore et al., 2000) and in the presence of other anti-bacterial agents (Greene et al., 1993). The quantified PAA ozone content reported in Figure 8 is supported by the atomic oxygen peaks (777 and 844 nm) observed in the OES spectra (Figure 3). This reactive oxygen species is involved in the formation of ozone in atmospheric air (Azyazov and Heaven, 2015; Wanjala et al., 2018).

## 5 Conclusion

The use of 3D printing in the additive manufacturing and rapid prototyping of this PAA setup allowed us to better assess its chemical and anti-bacterial capacity. The use of 3D printing technology expedites the development of PAA setups and allows the further understanding of the chemical interactions occurring between reactive species and aerosols within atmospheric non-thermal plasmas. Even though OH radicals and hydrogen peroxide were not observed, the device produced PAAs with very low pH and considerable amounts of nitrite and ozone. These allowed it to produce significant bactericidal surface effects against *E. coli*, *P. aeruginosa*, *S. aureus* and *S. enterica* serovar Abony. The rapid prototyping approach allows PAA devices to be easily improved. This could enable it to obtain a higher formation of hydroxyl radicals and hydrogen peroxide, increasing the potential of PAAs for disinfection purposes. Further research is required to understand chemical reactions occurring across gas and water PAA phases.

## Data Availability

The original contributions presented in the study are included in the article/Supplementary material, further inquiries can be directed to the corresponding author.
